# The Increase of Hospitals in Atlanta

**Published:** 1903-05

**Authors:** 


### EDITORIAL NOTES AND COMMENTS.

The Business office of THE JOURNAL-RECORD is No. 64 Marietta Street.

The Editorial office is 318-319-320 The Prudential.

Address all Business communications to Dr. M. B. Hutchins, Mgr.

Make remittances payable to THE ATLANTA JOURNAL-RECORD OF MEDICINE.

On matters pertaining to the Editorial and Original Communications address Dr. Bernard Wolff, Atlanta.

Reprints of original articles will be furnished at cost price. Requests for the same should always be made *on the manuscript.*

We will present, post-paid, on request, to each contributor of an original article, twenty (20) marked copies of THE JOURNAL-RECORD containing such article.

### THE INCREASE OF HOSPITALS IN ATLANTA.

THE notable deficiency in the matter of hospitals in this city which existed for a long time gives promise of being filled to overflowing. Besides several private and semi-private institutions, a small and struggling hospital for incurables, two denominational hospitals already in existence, the Methodists have in contemplation the establishment in the near future of a large hospital. While there is certainly no greater or more laudable charity than the maintenance of establishments for the care of the sick poor, there is such a thing as straining the quality of mercy. Too many hospitals not only engender the hospital habit among a certain class of the public, but it works a hardship upon members of the medical profession who depend for their support upon the class of patients most likely to patronize the hospitals. It is often very difficult to discriminate between a real and a spurious object of charity, and a free hospital opens the door to the practice of much deception and misstatement on the part of those seeking its benefits. We do not care to be rushed into the dispensary nuisance which is so prevalent and so baneful in its effects in some of the larger cities. As all such institutions are dependent upon the doctors for their maintenance and continuance, the latter should use proper judgment and forethought before lending their aid and influence to schemes that might prove of more harm to the profession to which they belong than of good to the public.

What is really and urgently needed by the city is a reception or isolation hospital for the care of contagious diseases. There is no place in the city where a case of any communicable disease other than smallpox will be admitted.

